# Internalised stigma in people with rheumatoid arthritis: a cross sectional study to establish the psychometric properties of the ISMI-RA

**DOI:** 10.1186/s12891-016-1089-5

**Published:** 2016-06-02

**Authors:** Elizabeth Corker, R. Claire Henderson, Heidi Lempp, June S. L. Brown

**Affiliations:** Psychology Department, Institute of Psychiatry, Psychology and Neuroscience, King’s College London, London, SE5 8AF UK; Health Service and Population Research Department, Institute of Psychiatry, Psychology and Neuroscience, King’s College London, London, UK; Academic Rheumatology, Faculty of Life Sciences & Medicine, King’s College London, London, UK

**Keywords:** Internalised stigma, Rheumatoid Arthritis, Methodology

## Abstract

**Background:**

Internalised stigma is theorized to be the internalisation and legitimisation of stereotypes of the diagnosis held in society and has not been quantified within patients with Rheumatoid Arthritis. This study aimed to: validate a modified version of a measure of internalised stigma, (the Internalised Stigma of Mental Illness scale, ISMI) for use in a group of patients diagnosed with rheumatoid arthritis; establish the consistency of the construct being measured, and to explore the levels of internalised stigma within this group.

**Methods:**

A cross-sectional survey was conducted in London, UK with participants receiving out-patient treatment for Rheumatoid Arthritis. Participants completed the ISMI-Rheumatoid Arthritis (ISMI-RA) and a measure of self-esteem.

**Results:**

One hundred respondents were interviewed by phone. The ISMI-RA was found to be reliable using a measure of internal consistency (α = 0.85) showed concurrent validity with the Index of Self Esteem (*r* = 0.58, *p* < 0.01) and discriminant validity with no association with gender (t = 1.43, *p* = 0.61). A quarter of respondents reported internalised stigma to a ‘severe’ level. Acceptability and feasibility were established. A confirmatory factor analysis provided some support for the model of internalised stigma.

**Conclusions:**

The application of the ISMI-RA among the Rheumatoid Arthritis population looks promising. Internalised stigma was found to be present within this group. More research is needed to generalize these results and to explore the effects of internalised stigma on treatment adherence and quality of life.

## Background

Rheumatoid Arthritis (RA) is a long-term inflammatory autoimmune disease. RA affects joints and tendons, and commonly results in joint stiffness, swelling and soreness [1]. It is estimated that around 1 % of the UK adult population have RA [2]. The female to male ratio is 3:1 [2–4] and onset is most likely in early middle age [2, 5]. Finally, RA has been found to be more common in people from a European, as opposed to an Asian background [3, 6].

RA also affects mortality. Although those diagnosed mainly die of similar causes compared to the general population, this usually happens three to ten years earlier than expected [7–9], mostly due to cardiovascular disease [1].

Against a background of long term disability when living with RA, stigma has also been identified as a social phenomenon amongst this population [4, 10]. Recent research has begun to differentiate between specific types of stigma. Firstly, public (or perceived) stigma which refers to the attitudes and behaviour of other people towards those with the stigmatised diagnosis or identity [11]. One study has found evidence of perceived stigma in people with a diagnosis of RA [10], whilst in another, participants reported feeling publically stigmatised because of their RA diagnosis and associated problems such as deformities and walking slowly [4]. Secondly, self stigma, (also known as internalised stigma), is defined as the awareness, legitimisation and application of societal stereotypes of the particular stigmatised diagnosis to the self, and may result in decreased self-esteem and self-efficacy [12]. In terms of public stigma, some long term health diagnosis are linked to a stigmatised identity [13] and can cause discrimination in many areas of life, such as healthcare, employment, education and personal relationships [14–16]. Additionally, negative attitudes are held towards those who have a physical disability that is caused by an illness, rather than an injury [17]. These stigmatising attitudes have been described as more difficult to deal with than the illness itself [18]. Relatively high levels of public stigma have been reported in RA [10].

With regards to internalised stigma, the focus of research was initially on people who live with a mental illness diagnosis. The Internalised Stigma of Mental Illness (ISMI) scale is commonly utilized to measure this construct [19]. However, the ISMI has now been validated for use in some physical health populations [20], including leprosy [21, 22], HIV/AIDS [22], irritable bowel syndrome [23] and epilepsy [24]. Available psychometric data for these modifications suggest that these adapted measures are reliable in these groups [21, 22] and demonstrate that a third of a stigmatized population will report moderate or high internalised stigma [23, 25]. High levels of internalized stigma, are associated with low degree of self esteem [12], help seeking [26] and treatment adherence [27].

Psychometric validation however, is limited with the ISMI. The authors found only one study, based on a sample with mental illness, which studied construct validity using Factor Analysis and measurement invariance [28], ensuring that the same construct, that is, internalised stigma has being measured across time.

The existence of internalised stigma in people with a diagnosis of RA has not been quantified. The impact of RA on identity, and concerns about stigma have been explored in a qualitative study [4]. This study found that people with RA have to reconcile their previous roles within private and public spheres with their new identity as a person diagnosed with RA. This is seen to cause emotional distress and role conflicts, such as when a formerly independent person or someone who has been a parent or carer then has to accept help from others.

The aims of this study were threefold. Firstly, to validate a modified version of the ISMI in people with a diagnosis of RA (to be called ISMI-RA), and establish (i) its reliability; (ii) its concurrent validity; (iii) its discriminant validity and to determine (iv) its acceptability and (v) feasibility. The second aim is to further validate the ISMI-RA and carry out a confirmatory factor analysis (CFA) to establish the consistency of the construct being measured. Finally, we wanted to explore levels of internalised stigma, using the ISMI-RA, within an RA outpatient clinic population. Given the previous literature regarding internalised stigma in various health conditions, we predicted that internalised stigma would be present in levels above ‘minimal’ [29] in around 30 % of this RA group.

## Methods

A cross-sectional study was conducted between January 2013 and September 2013 in London, UK. A convenience sample was chosen to recruit participants from two hospitals in London. The inclusion criteria were: (i) attendance of a rheumatology outpatient clinic at one of the two sites; (ii) had received a diagnosis of RA (early or established) by a consultant rheumatologist; (iii) aged 16–65; (iv) English speaking, and (v) were not an inpatient at the time of the study. Patients who were diagnosed before 2010 (*n* = 77) met the American College of Rheumatology criteria for RA [30], whereas those diagnosed after 2010 (*n* = 23) met the revised American College of Rheumatology and European League against Rheumatism criteria for RA [31]. Potential participants were identified by a research nurse, who screened for eligibility and briefly explained the project after a routine appointment. If patients expressed an interest to take part, the researcher then explained the project in more detail and gave them an envelope, which included information about the study, a consent form and sources of additional support. Outpatient attendees took the documents away to read and return the signed consent form to the researcher once they were ready to take part and agreed to be interviewed by telephone.

### Instruments

#### The internalised stigma of mental illness scale

The Internalised Stigma of Mental Illness scale (ISMI) [19] was developed to measure the subjective experience of stigma in collaboration with mental health service users. Five subscales (alienation, stereotype endorsement, perceived discrimination, social withdrawal and stigma resistance) with a total of 29 items are rated by participants on a four point Likert scale. The scale has good internal reliability in a sample of people with psychiatric illnesses (α = 0.90) and test-retest reliability (*r* = 0.92). For the present study, all references to ‘mental illness’ were replaced with ‘rheumatoid arthritis’. Additionally, one item, “people with mental illness tend to be violent” was removed. Permission to modify the ISMI was granted from the authors. Finally, as recommended in previous studies, the stigma resistance subscale was removed from the analysis, as it has been found to be a separate construct to internalized stigma [28, 32]. Higher scores indicate higher internalised stigma: a score between 1.00 and 2.00 indicates minimal or no internalised stigma; 2.01–2.50 indicates mild internalised stigma, 2.51–3.00 indicates moderate internalised stigma and 3.01–4.00 indicates severe internalised stigma [29]. A two category scoring system is used by the author of the scale in a review of the use of the ISMI, in which scores below 2.50 are classified as ‘low internalised stigma’ and scores equal to or above 2.51 are classified as ‘high internalised stigma’ [20], this scoring system was used in the present analysis.

#### The index of self esteem

The Index of Self Esteem (ISE) [33] measures the self evaluative aspect of self- esteem. Participants rate 25 items that relate to self- esteem on a seven point Likert scale with a total possible score of 100. The Walmyr Assessment Scales Scoring Manual was used to categorise the raw scores. Higher scores indicate lower self-esteem. A score of 30 and above indicates self esteem difficulties and scores over 70 indicate severe self- esteem problems.

### Statistics

Data were entered into IBM SPSS (v.20). The confirmatory factor analysis was conducted using AMOS (v.20); all other analyses were conducted using IBM SPSS (v. 20).

Internal reliability was established to determine Cronbach’s alpha’s for each ISMI-RA subscale and the total score. Concurrent validity was tested with a Pearson’s correlation between the total ISMI-RA and subscale scores and the ISE. Although the ISE measures self- esteem which would be theorised to have a negative association with internalised stigma, the scoring of the ISE is such that higher score indicates low self esteem, therefore the direction of the association is appropriate for a concurrent validity test. This variable was selected as a measure of divergent validity as it is expected that ISMI-RA scores will not have a significant association with gender. No significant difference in ISMI-RA scores by gender will be taken as the appropriate criterion. Feasibility was assessed by timing the duration to complete the ISMI-RA. Over 30 min (mean average) is considered to be too long for a survey measure [34]. Acceptability was examined by measuring floor and ceiling effects that would violate Maximum Endorsement Frequencies (MEF); if more than 15 % of participants scored in the highest and lowest categories, this would be a violation of MEF [35]. A CFA was performed to study the factor structure of the latent construct of internalised stigma [36]. A CFA was deemed an appropriate test for model fit, as the manifest variables have been previously defined in the literature, and the present study aimed to assess these variables with regards to an RA population. Manifest variables in the model were Alienation, Stereotype Endorsement, Discrimination Experience and Social Withdrawal, the latent construct was self stigma. Model fit was assessed with multiple fit statistics; the goodness of fit index (GFI), the root mean square error of approximation (RMSEA) and the goodness of fit chi-square. Model fit would be supported if the GFI, RMSEA and goodness of fit did not reach statistical significance.

## Results

A total of 100 respondents were included in the study; 25 males and 75 females (see Table 1). A total of 127 out patients eligible to participate were approached for consent, 27 out patients declined to take part, yielding a response rate of 79 %. Reasons for non-participation were not sought. The mean age of the group was 46.3 years (SD: 10.9; range 23–65). The majority of the sample self-described themselves as white British (75.2 %) and over half were either in work full time (36.6 %), or part time (27.7 %). The mean number of years since the first contact with RA services was 6.0 years (SD: 6.1; range 1–31).

**Table 1:** Socio-demographic characteristics of participants

Characteristic	Participants (*n* = 100)
Gender	n (%)
Male	25 (25.0)
Female	75 (75.0)
Age (years)	
Range	23–65
Mean (SD)	46.3 (10.9)
Years since first contact with RA services	
Range	1–31
Median (SD)	6.0 (6.1)
Ethnicity	n (%)
White British	76 (76.0)
Other White	9 (9.0)
Black or Mixed Black & White	7 (7.0)
Asian or Mixed Asian & White	5 (5.0)
Other Mixed	0
Other	1 (1.0)
Did not wish to disclose	2 (2.0)
Employment status	n (%)
Unemployed	8 (8.0)
Part-time employed	28 (28.0)
Full-time employed	37 (37.0)
Retired	23 (23.0)
Volunteering	0
Training/education	2 (2.0)
Other (incl. Self-employed)	2 (2.0)

### The internalised stigma of mental illness scale

Overall, 25 % of the sample reported moderate or severe levels of internalised stigma. A further 49 % reported mild levels of internalised stigma. As summarised in Table 2, the mean total score for the ISMI-RA was 2.2 (SD: 0.36; range: 1.04–3.13). This is comparable to scores found in participants with HIV/AIDS (2.3) and leprosy (2.2) [22]. ISMI-RA subscale scores are shown in Table 2. Mean scores ranged from 2.0 (stereotype endorsement) to 2.7 (alienation).

**Table 2:** Measures of reliability for ISMI-RA

Measure	Mean (SD)	Range	Internal consistency (α)	Top/bottom scores (% of pts)	
ISMI				Top	Bottom
Total	2.2 (0.36)	1.04–3.13	0.85	1	1
Alienation	2.7 (0.61)	1.17–4.00	0.90	1	2
Stereotype endorsement	2.0 (0.26)	1.00–2.67	0.49	2	1
Social withdrawal	2.2 (0.45)	1.00–3.33	0.82	1	1
Discrimination experience	2.1 (0.32)	1.00–3.00	0.65	2	1
ISE	36.8	12.0–60.7	0.95	2	1

#### Profile of internalised stigma

Frequency of agreement of items ranged from 0 % (people with RA should not get married) to 77 % (people without RA could not possibly understand me). The most frequently endorsed (that is, agreed with) items were: people without RA could not possibly understand me (77 %); I am disappointed in myself for having RA (67 %); I am embarrassed or ashamed that I have RA (56 %); I feel inferior to others who don’t have RA (52 %) and people can tell that I have RA by the way I look (51.5 %), see Table 3 for further details. Four of the five most frequently endorsed items belonged to the alienation subscale, the remaining one belonged to the stereotype endorsement subscale.

**Table 3:** Endorsement frequencies and completeness of ISMI-RA items

ISMI-RA item	Response	*n* (%)
I feel out of place in the world because I have RA	Disagree	60 (60.0)
	Agree	40 (40.0)
	Missing	0
People discriminate against me because I have RA	Disagree	80 (80.0)
	Agree	20 (20.0)
	Missing	0
I avoid getting close to people who don’t have RA to avoid rejection	Disagree	77 (77.0)
	Agree	23 (23.0)
	Missing	0
I am embarrassed or ashamed that I have RA	Disagree	44 (44.0)
	Agree	56 (56.0)
	Missing	0
People with RA should not get married	Disagree	100 (100.0)
	Agree	0
	Missing	0
People with RA make important contributions to society	Disagree	14 (14.0)
	Agree	86 (86.0)
	Missing	0
People with RA make important contributions to society	Disagree	14 (14.0)
	Agree	86 (86.0)
	Missing	0
I feel inferior to others who don’t have RA	Disagree	48 (48.0)
	Agree	52 (52.0)
	Missing	0
I do not socialize as much as I used to because my RA might make me look or behave in a “strange” way	Disagree	69 (69.0)
	Agree	31 (31.0)
	Missing	0
People with RA cannot live a good, rewarding life	Disagree	80 (80.0)
	Agree	20 (20.0)
	Missing	0
Negative stereotypes against people with RA like myself keep me isolated from the “normal” world	Disagree	81(81.0)
	Agree	19 (19.0)
	Missing	0
Being around people who do not have RA makes me feel out of place or inadequate	Disagree	49 (49.0)
	Agree	51 (51.0)
	Missing	0
I feel comfortable being seen in public who obviously has RA	Disagree	13 (49.0)
	Agree	87 (87.0)
	Missing	0
People often patronise me, or treat me like a child, just because I have RA	Disagree	84 (84.0)
	Agree	16 (16.0)
	Missing	0
I am disappointed in myself for having RA	Disagree	33 (33.0)
	Agree	67 (67.0)
	Missing	0
Having RA has spoiled my life	Disagree	51 (51.0)
	Agree	49 (49.0)
	Missing	0
People can tell that I have RA by the way I look	Disagree	48 (48.5)
	Agree	51 (51.5)
	Missing	1
Because I have RA, I need others to make most decisions for me	Disagree	97 (97.0)
	Agree	3 (3.0)
	Missing	0
I stay away from social situations in order to protect my family or friends from embarrassment	Disagree	74 (74.0)
	Agree	26 (26.0)
	Missing	0
People without RA could not possibly understand me	Disagree	23 (23.0)
	Agree	77 (77.0)
	Missing	0
People ignore me or take me less seriously just because I have RA	Disagree	89 (89.0)
	Agree	11 (11.0)
	Missing	0
I cannot contribute anything to society because I have RA	Disagree	82 (82.0)
	Agree	18 (18.0)
	Missing	0
Living with RA has made me a tough survivor	Disagree	58 (58.0)
	Agree	42 (42.0)
	Missing	0
Nobody would be interested in getting close to me because I have RA	Disagree	91 (91.0)
	Agree	9 (9.0)
	Missing	0
In general, I am able to live life the way I want to	Disagree	29 (29.0)
	Agree	71 (71.0)
	Missing	0
I can have a good, fulfilling life, despite my RA	Disagree	23 (29.0)
	Agree	77 (77.0)
	Missing	0
Others think that I cannot achieve much in life because I have RA	Disagree	85 (85.9)
	Agree	14 (14.1)
	Missing	1
Stereotypes about RA apply to me	Disagree	87 (87.0)
	Agree	13 (13.0)
	Missing	0
I don’t talk about myself much because I don’t want to burden others with my RA	Disagree	77 (77.0)
	Agree	23 (23.0)
	Missing	0

By contrast, the least frequently endorsed items were: people with RA should not get married (0 %); because I have RA, I need others to make most decisions for me (3 %); nobody would be interested in getting close to me because I have RA (9 %); people ignore me, or take me less seriously because I have RA (11 %) and stereotypes about people with RA apply to me (13 %). Three of the five least frequently endorsed items belonged to the stereotype endorsement subscale, the remaining two from the discrimination experience subscale.

#### The index of self esteem

Table 2 illustrates the mean total score for the ISE was 36.8 (SD: 10.2; range: 12.0–60.7) and had an internal consistency of α = 0.95.

#### Reliability of ISMI-RA

The Cronbach’s alpha value indicating internal consistency for the total ISMI-RA was α = 0.85. The subscales ranged from α = 0.49 (stereotype endorsement) to α = 0.90 (alienation), see Table 2. All alpha values were within the accepted range for internal consistency [37, 38].

#### Acceptability of ISMI-RA

Fewer than 15 % of respondents scored in the bottom or top categories of the ISMI- RA total or any of the subscales (see Table 2), which demonstrates a lack of floor or ceiling effects respectively using MEF assumptions [35].

#### Feasibility of ISMI-RA

The mean time to complete the ISMI-RA was 8 min, which is considered an acceptable duration. Twenty six out of 28 items contained no missing data, for the remaining two items, one participant’s data was missing.

#### Concurrent validity

A Pearson’s correlation revealed a significant, positive correlation between the total ISMI-RA score and the ISE score (*r =* 0.58, *p* < 0.01). Significant, positive correlations were also found between the ISE and all ISMI-RA subscales: stereotype endorsement and the ISE (*r =* 0.48, *p* < 0.01); alienation and the ISE (*r =* 0.48, *p* < 0.01); discrimination experience and the ISE (*r =* 0.48, *p* < 0.01) and social withdrawal and the ISE (*r =* 0.48, *p* < 0.01).

#### Confirmatory factor analysis

**Fig. 1:**
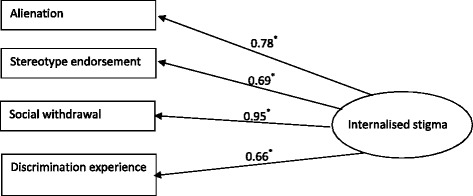
Confirmatory factor analysis model

The path diagram with factor loading (standard regression weights) is displayed in Fig. 1. All manifest variables (rectangles in Fig. 1) were found to be good indicators of the latent factor (internalised stigma, displayed as an oval in Fig. 1), weights ranged from 0.63 to 0.96, all *p* values were <0.001. The overall model had a chi-square value of 11.2 (df = 2), *p* = 0.004.

However, the goodness-of-fit test was significant, which does not support the fit between our hypothesised model and the data. On the other hand, the GFI provided support for model fit (0.91) but the RMSEA did not (0.29).

## Discussion

Similar to previous studies that have modified the ISMI for leprosy, HIV/AIDS and inflammatory bowel disease [21–23], we have found that the modified version of the ISMI is probably acceptable for use in patients with a diagnosis of Rheumatoid Arthritis: all Cronbach’s alpha’s were acceptable; there were no floor or ceiling effects; the duration of administration of the measure was feasible; concurrent validity was found with self-esteem; discriminant validity was found with a lack of association with gender. Although concurrent validity was assessed with a measure of self- esteem, we are confident that because of the direction of scoring with the ISE, that this provides the positive association required for establishing concurrent validity. With regards to the second aim, the CFA provided some support that the subscales were indices of one factor, the latent construct, internalised stigma even though not all the goodness of fit measures supported the model. As a CFA usually assumes multidimensionality, and self stigma has been suggested to be a construct containing different dimensions [39], this was an appropriate test for model fit. Finally, we wanted to explore levels of internalised stigma within an RA outpatient population. The results showed that 25 % of the group displayed severe or moderate levels of internalised stigma and a further 49 % displayed a mild level. This was a higher proportion than our prediction that internalised stigma would be present in around 30 % of this RA group. Additionally, the mean scores of the total ISMI-RA were comparable to previous studies that explored levels of internalised stigma in HIV/AIDS, leprosy and mental illness [22, 40]. Our results support previous findings, that suggest that people with a long term health diagnosis experience stigma and discrimination [13–16], and that people diagnosed with RA specifically encounter stigma and discrimination [10]; 20 % of participants reporting that other people had discriminated against them, and 17 % feeling patronized because of their RA.

We expected stereotype endorsement to be a key feature in the profile of the ISMI-RA results, as this has been theorized to be a major component of internalised stigma, [12, 41, 42]. However, the mean score for the stereotype endorsement subscale was 2.0, which falls within the ‘mild’ internalised stigma classification [19]. Furthermore, this subscale had the lowest mean score. This pattern has been found in previous studies that report subscale scores, for mental illness [43, 44] and inflammatory bowel disease [23]. Taken together, these results indicate that stereotype endorsement may not be the key component within self stigma in patients with RA. The highest subscale score in this study was alienation, which fell into the ‘moderate’ internalised stigma classification. In previous studies that focused on patients with a diagnoses of physical long-term conditions, the most endorsed subscale is usually found to be social withdrawal or alienation [23, 43, 44]. The current study supports this pattern.

The results from the current study support work conducted by Lempp and colleagues who found that having a diagnosis of RA impacted upon a person’s identity and their roles within society as well as their private lives [4]. Relevant items such as: “having RA has spoiled my life”, “I don’t socialize as much as I used to because of my RA” and, “I cannot contribute anything to society because I have RA”, were endorsed by sizable proportions of the sample (49 %, 31 % and 18 % respectively) and suggests that an RA diagnosis can impact on several different aspects of the participants’ lives.

### Strengths and limitations

This is the first study that has quantified internalised stigma in a group of people who live with early and established RA. Additionally, we have successfully modified the ISMI to extend the scale further within this patient group. The main limitation was within the CFA, with some of the fitness indices that indicated that the data was not a good fit of the model. In order to sufficiently power a CFA, it is recommended that data from at least 200 participants are analysed [45, 46]. Due to time and resource constraints, we were only able to interview 100 participants. We conclude however that due to the results of the other validation tests and the model analysis that the ISMI-RA is promising for application in a population with RA. Additionally, we would suggest that future studies assess the test-retest reliability of the ISMI-RA in order to assess for reliability of the scale over time.

Finally, all our participants were receiving care from a specialist out-patient NHS clinic in a relatively wealthy and ethnically diverse city in a high income country. Our results may therefore not be generalisable to people who receive services in middle or low income countries or do not receive any services at all. It may be reasonable to predict that these populations would report higher internalised stigma related to their RA. On the other hand, it may also be the case that in places where the treatment of RA is not as developed that people diagnosed with RA may not conceptualise it as part of their identity and therefore not experience internalised stigma [42] to the same degree as identified in this study. Unfortunately, we do not know how our sample differed from people who did not take part. The original ISMI was developed in a Veterans Administration hospital within the US in an outpatient clinic population with mental illness, the majority of whom were male (Ritsher et al. [19]). Since its inception, the ISMI has been adapted for use across a range of illnesses, languages and cultures, and has been found to be reliable and valid [20].

#### Further research

Generalization could be improved by recruiting participants from a selection of urban and rural locations. Additional measures could also be utilized to explore the effect of internalised stigma on treatment adherence, perceptions of disability and quality of life.

Patients who received a diagnosis of RA before the introduction of new treatment options such as biologics tend to have more visible deformities and therefore may experience more stigma and discrimination [4] and we suggest that further work needs to explore the effects of visible deformities in internalised stigma.

We would recommend that the item “people with RA should not get married” be removed from future work using the ISMI-RA as this item was not endorsed by any participant. We would also recommend a Rasch analysis be conducted on future ISMI or ISMI-RA data, in order to test for justification in adding scores to create a total.

At present, the authors are conducting a qualitative study with a subsample of the current participants to examine which aspects of internalised stigma impact on participants’ lives and identity. We hope this will also provide further validation for the use of the ISMI-RA.

Finally, the authors are also comparing a sample of patients with a diagnosis RA with a sample of people who receive care for a psychiatric illness, due to their previously confirmed experienced internalised stigma [19, 43, 44, 47–49]. It would be expected from the current and previous studies that internalised stigma related to a diagnosis of RA would exist in a comparable level to a psychiatric group and may open up new research avenues to investigate the effects of the diagnosis of other long term illnesses on identity and internalised stigma.

## Conclusions

Application of the ISMI-RA appears to be an encouraging way of measuring internalised stigma in this group. More research is required to improve the generalisability of these findings and to compare RA internalised stigma levels to other long term physical and/or mental illnesses groups.

## Abbreviations

ISMI, internalized stigma of mental illness scale; ISMI-RA, internalised stigma of mental illness scale, Rheumatoid Arthritis version; RA, Rheumatoid Arthritis
